# *Moringa oleifera* seeds-removed ripened pods as alternative for papersheet production: antimicrobial activity and their phytoconstituents profile using HPLC

**DOI:** 10.1038/s41598-021-98415-9

**Published:** 2021-09-24

**Authors:** Mohamed Z. M. Salem, Hayssam M. Ali, Mohammad Akrami

**Affiliations:** 1 Forestry and Wood Technology Department, Faculty of Agriculture (EL-Shatby), Alexandria University, Alexandria, 21545 Egypt; 2 Botany and Microbiology Department, College of Science, King Saud University, P.O. Box 2455, Riyadh, 11451 Saudi Arabia; 3 Department of Engineering, University of Exeter, Exeter, EX4 4QF UK

**Keywords:** Biological techniques, Environmental sciences, Engineering, Materials science

## Abstract

In the present study, and for the waste valorization, *Moringa oleifera* seeds-removed ripened pods (SRRP) were used for papersheet production and for the extraction of bioactive compounds. Fibers were characterized by SEM–EDX patterns, while the phytoconstituents in ethanol extract was analyzed by HPLC. The inhibition percentage of fungal mycelial growth (IFMG) of the treated *Melia azedarach* wood with *M. oleifera* SRRP extract at the concentrations of 10,000, 20,000, and 30,000 µg/mL against the growth of *Rhizoctonia solani* and *Fusarium culmorum* was calculated and compared with fluconazole (25 µg). The produced papersheet was treated with the ethanol extract (4000, 2000, and 1000 µg/mL) and assayed for its antibacterial activity against *Agrobacterium tumefaciens*, *Erwinia amylovora*, and *Pectobacterium atrosepticum* by measuring the inhibition zones and minimum inhibitory concentrations (MICs). According to chemical analysis of *M. oleifera* SRRP, benzene:alcohol extractives, holocellulose, lignin, and ash contents were 7.56, 64.94, 25.66 and 1.53%, respectively, while for the produced unbleached pulp, the screen pulp yield and the Kappa number were 39% and 25, respectively. The produced papersheet showed tensile index, tear index, burst index, and double fold number values of 58.8 N m/g, 3.38 mN m^2^/g, 3.86 kPa m^2^/g, and 10.66, respectively. SEM examination showed that the average fiber diameter was 16.39 µm, and the mass average of for elemental composition of C and O by EDX were, 44.21%, and 55.79%, respectively. The main phytoconstituents in the extract (mg/100 g extract) by HPLC were vanillic acid (5053.49), benzoic acid (262.98), naringenin (133.02), chlorogenic acid (66.16), and myricetin (56.27). After 14 days of incubation, *M. oleifera* SRRP extract-wood treated showed good IFMG against *R. solani* (36.88%) and *F. culmorum* (51.66%) compared to fluconazole, where it observed 42.96% and 53.70%, respectively. Moderate to significant antibacterial activity was found, where the minimum inhibitory concentration (MIC) values were 500, 650, and 250 µg/mL against the growth of *A. tumefaciens*, *E. amylovora*, and *P. atrosepticum* respectively, which were lower than the positive control used (Tobramycin 10 µg/disc). In conclusion, *M. oleifera* SRRP showed promising properties as a raw material for pulp and paper production as well as for the extraction of bioactive compounds.

## Introduction

*Moringa oleifera* Lam. (family Moringaceae) is a fast-growing and drought-resistant tree, native to the Indian subcontinent with multipurpose uses^1^. Fruits of Moringa are three-sided pods with pendulous and linear shape, also, the pod generally has 250–450 mm long contains approximately 20 globular seeds^2^. From the literature survey, all the works are concentrated in how to use leaves, flowers, pods and roots of *Moringa* in different purposes^3–5^. Leaves and seeds of *M. oleifera* are promised as a first stage in the treatment for waste waters^6–8^ or for coagulant of primary treatment of paper mill effluent^9^. Acid activated from *M. oleifera* leaf was also prepared, which act as a good alternative adsorbent for dyes and heavy metal recoveries from aqueous solutions^10^. Petals of *M. oleifera* were used as a mediated green synthesis of gold nanoparticles^11^. Leaves and other parts from the tree were used as a source for antimicrobial and antioxidant agents as well as for pharmaceutical purposes^12–14^. In livestock application, leaves and seeds of *M. oleifera* are used for animal nutrition, where they have many nutritional compounds such as oils, carbohydrates, vitamins, fatty acids, amino acids, lipid, minerals and other chemical compounds^5,15,16^.

Several bioactive compounds were isolated and identified from different parts of Moringa (leaves, seeds, bark, flowers, pods, and root) and were summarized in the review articles of Chhikara et al*.*^2^ and Trigo et al*.*^17^. Quercetin, myricetin glycosides, caffeoylquinic acid, coumaroylquinic acid, hydroxybenzoic acid, kaempferol, glucotropaeolin, glucosinalbin, glucoraphanin, glucomoringin, glucoiberin, glucosinolates, apigenin, luteolin, lutein, luteoxanthin, zeaxanthin, b-carotene and isothiocyonates were identified as the main compounds in the extracts from moringa^2,18,19^. Phenolic compounds from *M. oleifera* seed, including gallic acid, ellagic acid and kaempferol were observed good antioxidant activity^20,21^.

For the production of pulp and paper from *M. oleifera*, there are little works from the literature, i.e., kraft pulping yield of *M. oleifera* and *M. concenensis* (*M. concanensis*) stems showed satisfactory strength properties for wrapping and writing papers compared to those of conventional raw materials^22^. Also, some investigations showed that the fiber characterizations such as fiber length and diameter of *M. oleifera* stem indicated that stem-wood from the middle and base was best suited for pulp and paper production^23^, while among the collected stems from 1, 3 and 5 year olds *Moringa oleifera*, the fiber characteristics from 5 year old *M. oleifera* stem-wood showed the best suited for the production of pulp and paper^24^.

To the best of our knowledge, this is the first work showing the value-added of *M. oleifera* seeds-removed ripened pods in the production of papersheet and as source for bioactive compounds for antibacterial and antifungal activities.

## Materials and methods

### Plant material and extract preparation of *Moringa oleifera* seeds-removed ripened pods

This study is complied with relevant institutional, national, and international guidelines and legislation. This study does not contain any studies with human participants or animals performed by any of the authors, where *Moringa oleifera* Lam. seeds-removed ripened pods (SRRP) were collected from Alexandria, Egypt, 2020. The plant was identified at the Department of Forestry and Wood Technology, Faculty of Agriculture, Alexandria University and a sample was deposited (voucher number Zidan0077). The SRRPs were ground into powder and screened (size 40–60 mesh), and then 100 g of this powdered size were extracted with ethanol (200 mL) by soaking method for 3 days^25^, where every day it was agitated at least three times for 5 min, and it should be noted that every day the amount of ethanol was replaced with the another amount (200 mL), therefore we used 600 mL ethanol for three days extraction. The extracted material was filtrated using Whatman filter paper no. 1 to get rid of residues and the dissolved extract was concentrated by evaporating the solvent using the rotary evaporator.

### The antifungal activity of wood treated with *M. oleifera* (SRRP) extract

Two fungi *Fusarium culmorum* (Acc# MH352452), and *Rhizoctonia solani* (Acc# MH352450), were used for the bioassay^26–28^. *Melia azedarach* wood specimens (2 × 1 × 0.5 cm), that autoclaved (121 °C for 20 min) and left to cool, were treated with *M. oleifera* SRRP extract at the concentrations of 10,000, 20,000, and 30,000 µg/mL. Each wood sample was received 100 µL from each concentration of *M. oleifera* SRRP extract. Petri dishes contained PDA media were inoculated with 5 mm-disc diameter of each fungus and the treated wood samples were put directly over the media at the opposite side of the fungus disc^29,30^. The treated wood samples were compared with control treatment (autoclaved-untreated). The percentage of fungal inhibition was calculated with the formula of the inhibition percentage of fungal mycelial growth (IFMG %) = [(T_0_ − T_1_)/T_0_] × 100, where T_0_ and T_1_ are the average diameters (mm) of fungal colonies under the control and experimental treatments, respectively, after insuring that the growth of fungi in control treatment, the measurement was done according to the previous works^28–33^. The IFMG values were compared with the positive (25 µg of fluconazole) and negative (10% DMSO) controls^34^.

### HPLC analysis of extract

HPLC 1260 Infinity Agilent System (Agilent Technologies, Santa Clara, CA, USA) equipped with a Quaternary pump and a Zorbax Eclipse Plus C18 column (100 mm × 4.6 mm i.d.) operated at 30 °C was used to identify the phytochemical compounds in *M. oleifera* SRRP extract. Separation conditions can be found in previously published works^4,27,35–38^. The following standard phytochemical compounds with HPLC grade (Sigma-Aldrich, St. Louis, MO, USA) were used; catechol, *p*-hydroxy benzoic acid, caffeine, chlorogenic acid, vanillic acid, caffeic acid, syringic acid, vanillin, *p*-coumaric acid, ferulic acid, benzoic acid, rutin, ellagic acid, *o*-coumaric acid, salicylic acid, cinnamic acid, myricetin, quercitin, rosmarinic acid, naringenin and kaempferol.

### Chemical analysis of *M. oleifera* SRP and Kraft pulping

**Figure 1:**
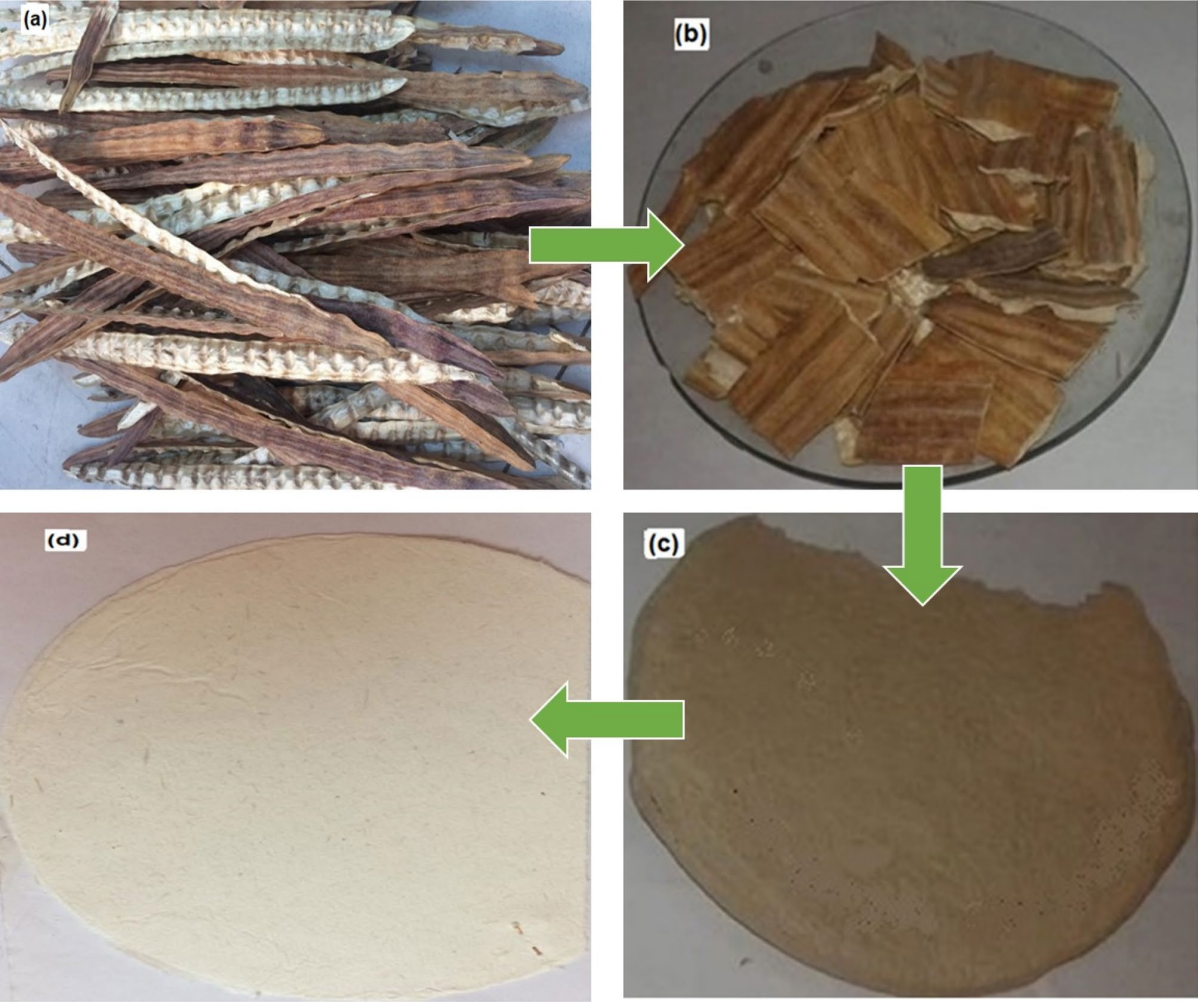
Shows *M. oleifera* SRRP (**a**), the cut pieces of *M. oleifera* SRRP (**b**), pulp of *M. oleifera* SRRP (**c**) and the produced papersheet from the pulp (**d**).

*Moringa oleifera* SRRP (Fig. 1a) was collected after the seeds were removed then cut into small pieces or flakes to be suitable for pulping (Fig. 1b). For chemical analysis, about 200 g of *M. oleifera* SRRP were ground into fine powder then screened to obtain the size 40–60 mesh fraction. Extractives content (alcohol and benzene), holocellulose, insoluble lignin content and Ash content were measured according to T204, T249, T222 om88, and T211, respectively.

For Kraft pulping**,** 200-g oven-dried pieces of *M. oleifera* SRRP were swelled for one day, filtrated, washed several time with hot water. Kraft pulping was conducted in stainless steel vessel with capacity 2 L under rotation in oil bath. The conditions used for pulping of *M. oleifera* SRRP were: active alkalinity (11%), temperature (160 °C), reaction time (35 min) and the liquor ratio (liquid to *M. oleifera* SRRP ratio of 10:1). The solid residue was defibrated, washes with hot and cold water till neutral pH, and the resulted pulp (Fig. 1c) screened in a valley flat screen having 0.25 mm slots. The screened unbleached pulp yield^39^, Kappa number of unbleached pulp^40^, the CSF Freeness of Pulp ^41^, and the Residual alkali^42^ were determined.

Papersheet forming (Fig. 1d) was carried out followed with our previous works, where the pulp with standard papersheet samples (200 cm^2^) with grammage of about 60 g/m^2^ were obtained^43^. Papersheets were made and tested for the strength properties according to TAPPI test methods T218 and T220. The papersheets were tested for tensile resistance T404, tear strength T414, bursting strength T405 and double fold T423. Analysis of physical strength of pulp was performed according to TAPPI standard methods with sheets standard 60 g/m^2^. All the experimental works were performed in triplicate.

### Examination of the produced paper sheets via scanning electron microscopy (SEM)

The produced papersheets (Fig. 1d) from *M. oleifera* SRRP pulp were studied by scanning electron microscopy (SEM), attached with energy dispersive spectrometry (EDX), JFC-1100E ion sputtering device (model JEOL/MP, JSM-IT200 Series, Japan) with acceleration voltage of 20.00 kV to show the elemental compotation and diameter of the fibers from three points and the average was taken. The measurements were taken from three parts of the paper sheets^25,44–46^.

### In vitro antibacterial evaluation of treated-papersheets with the extract

Discs with approximate dimension of 1 × 1 cm were cut from the *M. oleifera* SRRP pulp paper treated with three concentrations (4000, 2000, and 1000 µg/mL) from *M. oleifera* SRRP extract as well as the control treatment (DMSO 10%)^25^. Three plants pathogenic bacteria *Agrobacterium tumefaciens* (acc# MG706145), *Erwinia amylovora* (acc#LN876573) and *Pectobacterium atrosepticum* (acc#MG706146), were used for the antibacterial activity and were previously identified through molecular identification^47–51^. The agar disc diffusion method was employed for antibacterial activity determination of the extract by recording the inhibition zone^52^. All tests were performed in triplicate. Also, micro-dilution method with serial concentrations of 32–1000 µg/mL was measured and compared with the control (Tobramycin 10 µg/disc)^26,53^.

### Statistical analysis

Tensile index, burst index, tear index, double fold number, brightness and optical measurements from the tested papersheet produced from *M. oleifera* SRRP pulp paper were recorded as mean ± SD from three measurements. The measurements of antifungal and antibacterial activities were statistically analyzed with one way ANOVA using SAS system and comparisons among the means were recorded using LSD test at an alpha value of 0.05^54^.

### Compliance with ethical standards

This study is complied with relevant institutional, national, and international guidelines and legislation. “This study does not contain any studies with human participants or animals performed by any of the authors”.

## Results and discussion

### Chemical characterization of *M. oleifera* SRRP and unbleached pulp properties

Chemical characteristics of *M. oleifera* SRRP and the produced unbleached pulp are shown in Table 1. The level of holocellulose content in *M. oleifera* SRRP is 64.94%, which indicates that it would be good sources of cellulose and hemicellulose. Furthermore, this content is well-compared with those reported by other studies, where the holocellulose content in *M. oleifera* stem was 65.5%^55^. While it was lower than those from other non-woody materials, i.e.,* Sorghum bicolor* stalks (71.0%)^56^, *Musa sapientum* (73.43%), *M. paradisiaca* (72.60%) and *Tithonia diversifolia* (71.60%)^57^, bamboo (70.50%)^58^, Tunisian Alfa stems (68.2%)^59^, Date palm rachis (74.8%)^60,61^, *Hesperaloe funifera* (76.5%)^62^, Cotton stalks (72.9%)^63^, Canola straw (77.5%)^64^, *Luffa cylindrica* (83.0%)^65^, *Hibiscus cannabinus* (81.1%)^66^, *Arundo donax* (70.2%)^63^, and flax plant (70%)^46^. While it was higher than those from *Zea mays* stalks (62.33%) and *Sorghum bicolor* stalks (63.40%)^67^, lotus leaf stalks (53.8%)^68^, and *Posidonia oceanica* (61.8%)^60^.

**Table 1:** Chemical composition of *M. oleifera* SRRP and unbleached pulp.

Parameter measured	Value
**Chemical analysis of Moringa raw material**	
Benzene: alcohol extractives	7.56 ± 0.01% ^a^
Holocellulose	64.94 ± 0.01%
Insoluble lignin	25.66 ± 0.57%
Ash	1.53 ± 0.05%
**Unbleached pulp**	
Freeness	300 mL CSF
Screen pulp yield	39 ± 1%
Kappa number	25 ± 1
Residual alkali	13.4 ± 0.1 (g/L)

^a^ Values are presented as mean± SD. *SD* Standard deviation.

Comparing to the woody materials, holocellulose content in *M. oleifera* SRRP was lower than the amount presented in *Paulownia elongota* wood (75.74%)^69^, *Pinus pinaster* wood (69.6%)^63^, *Albizia lebbeck* wood (78.60%)^70^, *Eucalyptus globulus* wood (80.5%)^63^, *Acer rubrum* wood (67.4%)^71^, *Leucaena diversifolia* wood (77.9%)^63^, and depithed Bagasse (72.38%)^72^. While it was higher than from those of *Prosopis alba* wood (63.6%)^63^, *E. camaldulensis* wood (56%) and *Meryta sinclairii* wood-branch (61%)^25^, and woods from *Bougainvillea spectabilis* (54.56%), *Ficus altissima* (54.73%), and *F. elastica* (53.37%)^67^.

Lignin content (25.66%) in *M. oleifera* SRRRP was lower than from the reported in *M. oleifera* stem (20.5%)^55^. While it was equal to those found in lotus leaf stalks (25.4%)^68^, and were higher than those from rice husks (21.98%)^73^, rice hulls (20.44–23.33%)^74^, sugar beet (17.67%)^75^, stalks of *Zea mays* (19.9–20.1%)^76^, Sweet sorghum (21%)^77^, Corn stover (19%)^78^, Tall fescue (14.0%), and *Miscanthus giganteus* (17.8%)^79^, bamboo (24.5%)^58^, *H. funifera* (7.3%)^62^, Cotton stalks (21.4%)^63^, Canola straw (20.0%)^64^, *Luffa cylindrica* (15.2%)^65^, Kenaf (12.7%)^66^, Wheat straw (19.64%)^80^, *A. donax* (22.3%)^63^, flax plant (6.8%)^46^, depithed Bagasse (20.03%)^72^, Bagasse (23.33%)^81^, *Cynara cardunculus* stalks (16–19%)^82,83^ and *Miscanthus* × *giganteus* stalks (13%)^84^. While it was lower than amount from Nut shells (30–40%)^85^.

The content of lignin from *M. oleifera* SRRP was in the range of hardwood species (25–35%)^86^, i.e., in *Albizia lebbeck* wood (25.14%)^70^ and lower than those from Date palm rachis (27.2%)^49,61^, and *Posidonia oceanica* (29.8%)^60^. Compared to woody plant materials, it was lower than those from *Pinus pinaster* (26.2%)^63^, *Acer rubruma* (26.0%)^71^, and *E. camaldulensis* (27%)^25^, and higher than those from *E. globulus* (20.0%)^63^, *L. diversifolia* (19.1%)^63^, *P. alba* (19.3%)^63^, and *M. sinclairii* (23%)^25^.

The ash content in *M. oleifera* SRRP (1.53%) was lower than the amount in stem (3.5%)^55^, while the Alcohol-benzene solubility (7.56%) was higher from the measured in the stem (3.16%)^55^.

The unbleached *M. oleifera* SRRP pulp (Table 1) showed the following properties; Freeness (300 mL CSF), screen pulp yield (39%), Kappa number (25), and the residual alkali (13.4 g/L). Compared to other study, the screened yield from unbleached pulp of *M. oleifera* stem was 38.2–40.29%, Freeness mL, CSF (650), and Kappa number (16.2–21.7)^55^.

### Mechanical and optical properties of papersheets

Table 2 shows the mechanical and optical properties of the produced papersheet from *M. oleifera* SRRP pulp, where the tensile index (58.8 N m/g), tear index (3.38 mN m^2^/g), burst index (3.86 kPa m^2^/g), double fold number (10.66), brightness (32%) and opacity (67%).

**Table 2:** Mechanical and physical properties of the produced papersheet from *M. oleifera* SRRP pulp.

Mechanical properties				Optical properties	
Tensile index (N m/g)	Tear index (mN m^2^/g)	Burst index (kPa m^2^/g)	Double fold number (N)	Brightness (%)	Opacity (%)
58.8 ± 0.1 ^a^	3.38 ± 0.005	3.86 ± 0.01	10.66 ± 0.57	32 ± 1	67 ± 1

^a^ Values are presented as mean± SD. *SD* Standard deviation.

The tensile index value (58.8 N m/g) was higher than those reported from papersheet produced from rice straw pulps (38.0–55.2 N m/g)^87^, flax material (42.66 N m/g)^46^, and oil palm empty fruit bunches pulp (20.4 N m/g)^88^. While it was lower than from the papersheet produced from depithed Bagasse pulp (60 N m/g)^72^. The tear index value (3.38 mN m^2^/g) was lower than from papersheets manufactured from pulps of rice straw (6.49–7.49 mN m^2^/g)^87^, depithed Bagasse (5.0 mN m^2^/g)^72^, flax plant (4.33 mN m^2^/g)^46^ and palm oil empty fruit bunches (7.20 mN m^2^/g)^89^, while it was partially equal to the measured from wheat straw (3.86 mN m^2^/g)^90^ and higher than of sunflower stems (2.04 mN m^2^/g)^91^.

The burst index value (3.86 kPa m^2^/g) was in the range of the value reported from papersheets manufactured from pulps of rice straw (2.43–5.34 kPa m^2^/g)^87^, but lower than from depithed Bagasse (4.8 kPa m^2^/g)^72^. Double fold number (10.66) was lower than the value reported from the papersheets derived from pulps of rice straw (35–173)^87^, and depithed Bagasse (26–42)^72^.

Tensile, burst, and tear indices from papersheets produced from refined unbleached Kraft pulp from *M. oleifera* stem were 48.7 N m/g, 3.56 kPa m^2^/g, and 5.8 mN m^2^/g, respectively^55^. The unbleached pulp brightness of *M. oleifera* SRRP (32%) was higher than the reported from unbleached stem pulp (25.4–29.5%)^55^.

### SEM–EDX examination of the produced papersheet

**Figure 2:**
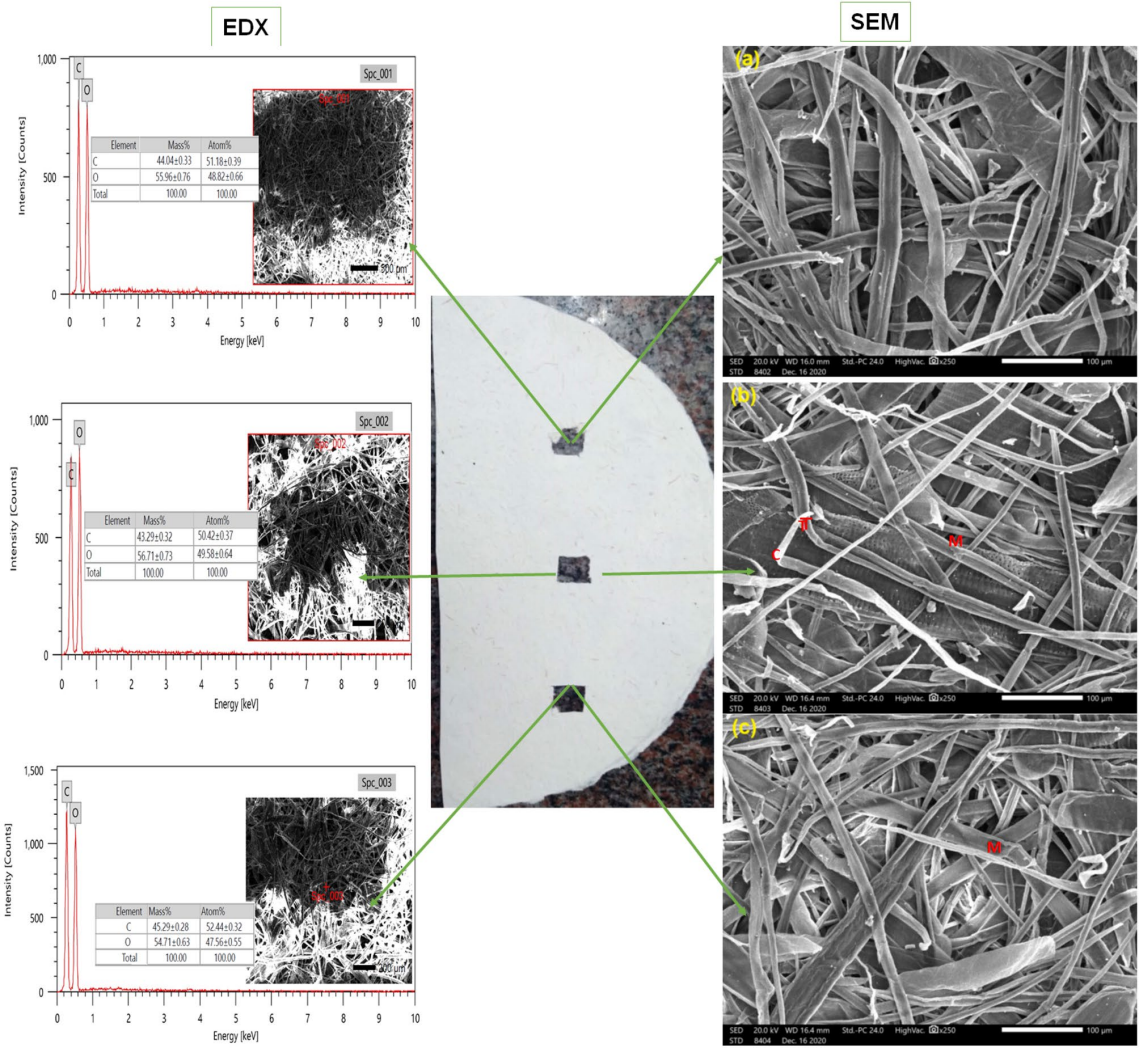
Shows the SEM–EDX measurements of papersheets from *M. oleifera* SRRP pulp paper at three points (**a**), (**b**) and (**c**). *C* Curl, *T* Twist, *M* Microcompression.

To confirm the distribution, construction and fiber diameters of the produced papersheet from *M. oleifera* SRRP pulp, SEM–EDX technique was used. The images of SEM–EDX were taken from three places of the produced papersheet. The SEM images showed that the average fiber diameters was 18.52 µm (Fig. 2a), 12.66 µm (Fig. 2b) and 18.29 µm (Fig. 2c), and the whole average was 16.39 µm. Other study designed to evaluate the fiber characteristics of *M. oleifera* wood slivers to predict its suitability for pulp and paper production showed that the average fibre diameter was 61.31 µm^23^, while other study showed that the value was 15.01 µm, 15.04 µm, and 15.08 µm from stem-wood of 1, 3, 5 years old *M. oleifera* trees, respectively^24^, and 15.0 μm in width^55^.

Furthermore, most of failure zones and the increase in fiber deformations, which probably could be found in pulp fibers such as curl, kink, lumen collapse, dislocation, microcompression and twist^92,93^ were shown in low amounts in *M. oleifera* SRRP papersheet.

Elemental composition by EDX showed that the mass (%) of C and O is 44.04%, 55.96% (Fig. 2 Spc_001), 43.29%, 56.71% (Fig. 2 Spc_002), and 45.29%, 54.71% (Fig. 2 Spc_003), and the mass average was 44.21 ± 1.01%, and 55.79 ± 1.01%, respectively.

### HPLC analysis, antibacterial and antifungal activities and extract from *M. oleifera* SRRP

**Figure 3:**
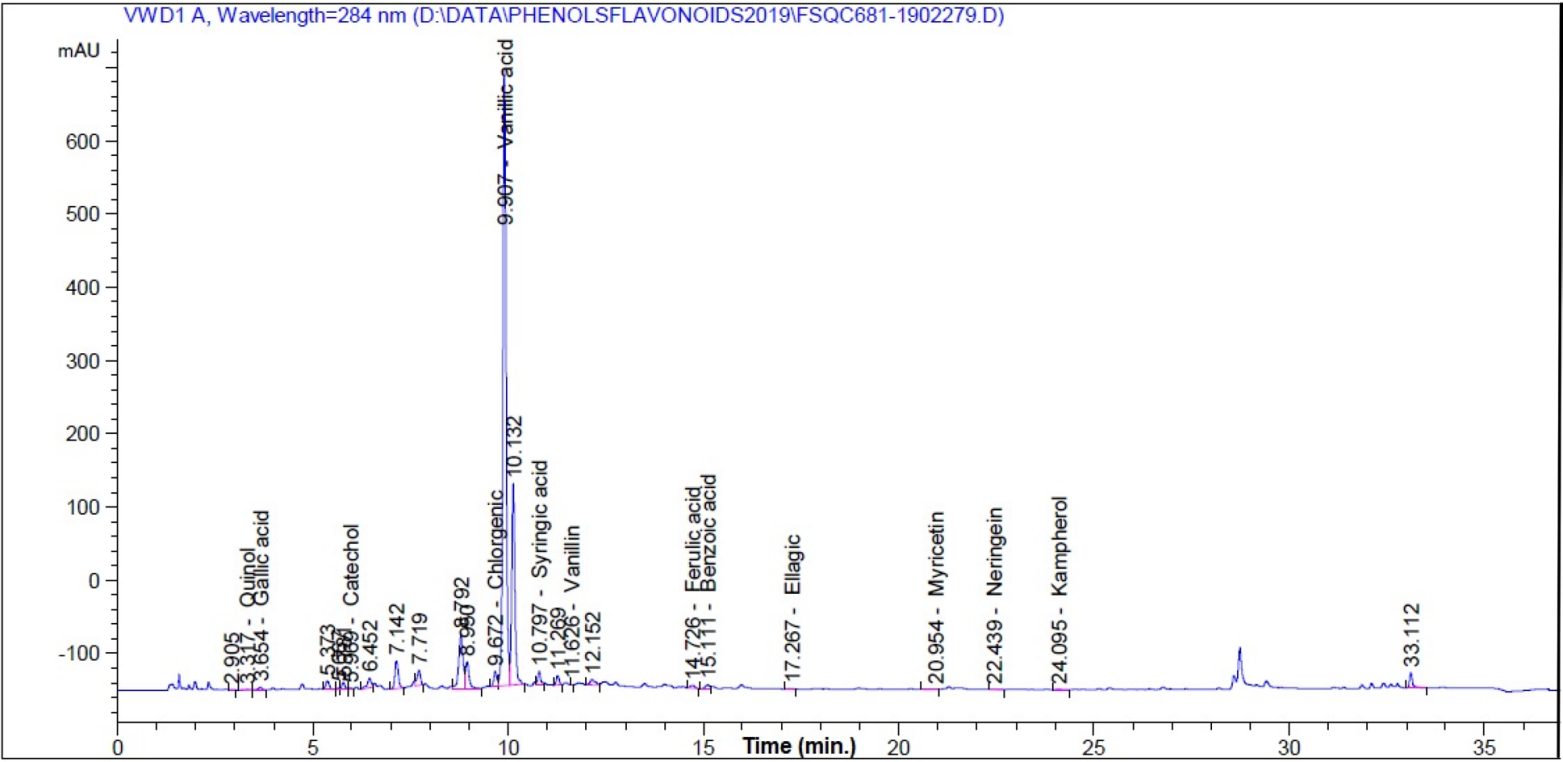
HPLC fingerprint of the identified phytoconstituents in *M. oleifera *SRRP extract.

Figure 3 shows the HPLC chromatogram of the polyphenolic compounds in the extract and the identified compounds is presented in Table 3, where the main compounds were vanillic acid (5053.49 mg/100 g extract), benzoic acid (262.98 mg/100 g extract), naringenin (133.02 mg/100 g extract), chlorogenic acid (66.16 mg/100 g extract), and myricetin (56.27 mg/100 g extract).

**Table 3:** Phytoconstituents profile of *M. oleifera* SRRP extract.

Compound	Amount (mg/100 g extract)
Catechol	20.85
*p*-Hydroxy benzoic acid	ND
Caffeine	ND
Chlorogenic acid	66.16
Vanillic acid	5053.49
Caffeic acid	ND
Syringic acid	48.029
Vanillin	0.849
*p*-Coumaric acid	ND
Ferulic acid	10.99
Benzoic acid	262.98
Rutin	ND
Ellagic acid	0.38
*o*-Coumaric acid	ND
Salicylic acid	ND
Cinnamic acid	ND
Myricetin	56.27
Quercitin	ND
Rosmarinic acid	ND
Naringenin	133.02
Kaempferol	31.71

*ND* Not detected.

**Figure 4:**
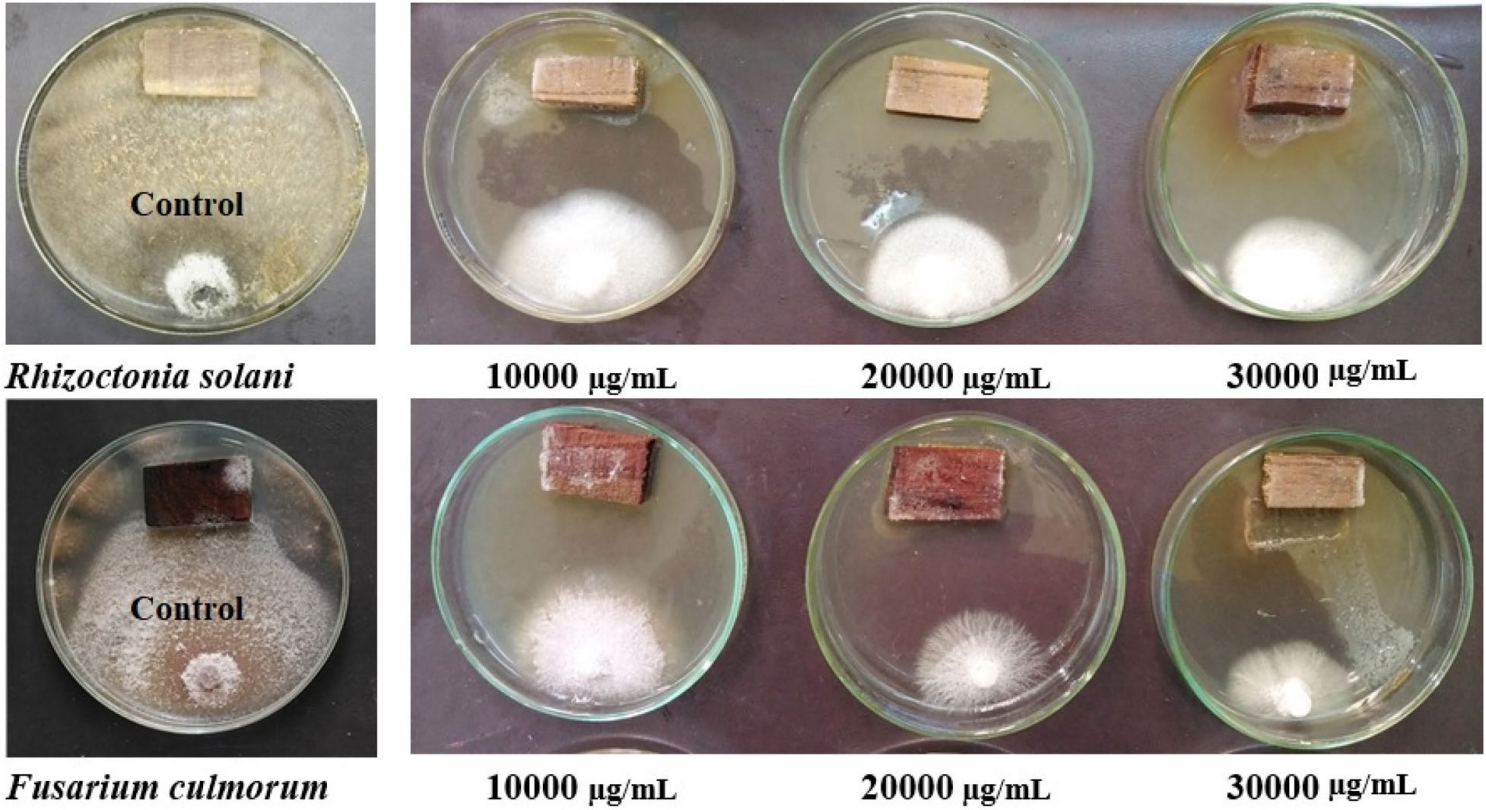
Visual observation after 14 days of the treated wood with *M. oleifera* SRRP extract and inoculated with two fungi (*Rhizoctonia solani* and *Fusarium culmorum*).

For the antifungal activity, the visual observations of wood-treated with *M. oleifera* SRRP extract and inoculated with *Rhizoctonia solani* and *Fusarium culmorum* after 14 days from the inoculation are shown in Fig. 4. Wood-treated with the extract showed inhibition percentage of fungal mycelial growth (IFMG) ranged from 27.51 to 36.88% and from 22.11 to 51.66% against the growth of *R. solani* and *F. culmorum*, respectively (Table 4).

**Table 4:** Antifungal activity of wood-treated *M. oleifera* SRRP extract.

Extract concentration (µg/mL)	Fungal mycelial inhibition percentage (%)	
	*Rhizoctonia solani*	*Fusarium culmorum*
10,000	27.51b ± 0.37^a^	22.11c ± 1.00
20,000	35.88a ± 0.33	30.66b ± 1.201
30,000	36.88a ± 0.66	51.66a ± 0.88
Control (10% DMSO)	0.00c	0.00d
Fluconazole (25 μg)^b^	42.96	53.70
LSD 0.05	1.36	2.92

Means with same letter within the same column are not significantly different according to LSD0.05.

^a^Values are presented as mean ± SE of fungal mycelial inhibition percentages.

^b^Data from our previous work^32^.

**Figure 5:**
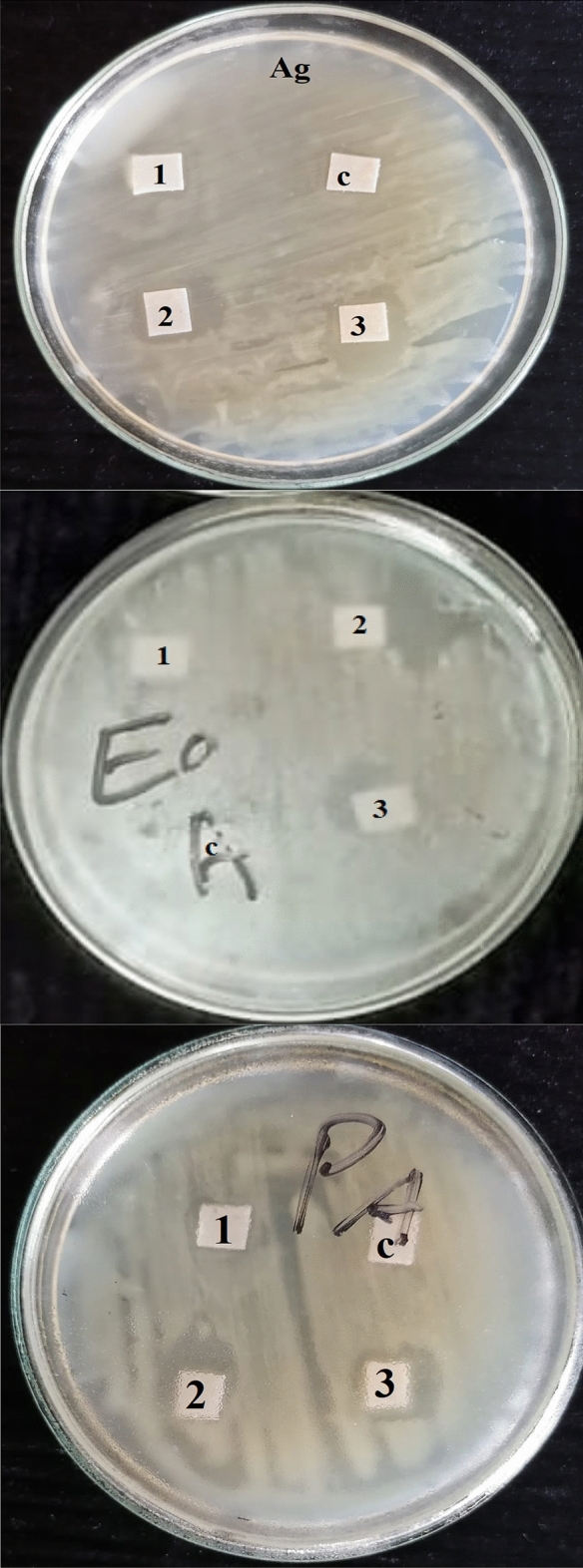
Antibacterial activity of treated papersheet discs with *M. oleifera* SRRP extract against (Ag) *Agrobacterium tumefaciens*; (Ea) *Erwinia amylovora*; (PA) *Pectobacterium atrosepticum*. *c*: Control; 1: Extract concentration 1000 µg/mL; 2: Extract concentration 2000 µg/mL; 3: Extract concentration 4000 µg/mL.

Table 5 observes that *M. oleifera* SRRP extract at 4000 µg/mL showed antibacterial activity against the growth of *Agrobacterium tumefaciens*, *Erwinia amylovora*, and *Pectobacterium atrosepticum*, with inhibition zones values of 11 mm, 6.66 mm and 16.66 mm, respectively, after the incubation period (24 h) as shown in Fig. 5. The recorded MIC values 500, 650, and 250 µg/mL against the growth of *A. tumefaciens*, *E. amylovora* and *P. atrosepticum*, respectively, were lower than of the positive control (Tobramycin 10 µg/disc) 32–64 µg/mL.

**Table 5:** Antibacterial activity of extract from *M. oleifera* SRRP.

Extract concentration (µg/mL)	Inhibition zones (cm)		
	*Agrobacterium tumefaciens*	*Erwinia amylovora*	*Pectobacterium atrosepticum*
4000	11a ± 1.00^*^	6.66a ± 2.08	16.66a ± 4.16
2000	8.66b ± 0.57	4.66a ± 2.08	15.33a ± 4.72
1000	2.66c ± 0.57	1.66b ± 0.57	2.33b ± 1.15
Control (10% DMSO)	0.00d	0.00b	0.00b
MIC (µg/mL)	500	650	250
MIC (Tobramycin 10 µg/disc) (µg/mL)	32	64	32
LSD 0.05	1.21	2.82	6.028
P-value	< 0.0001	0.0026	0.0003

Means with same letter within the same column are not significantly different according to LSD0.05.

*MIC* Minimum inhibitory concentration (µg/mL).

^*^Values are presented as mean ± SE of the inhibition zones.

It is important to note that a MIC value between 100 and 200 μg/mL was considered as positive for plant extracts^94–98^. However, the activity of plant extracts have been classified as significant (MIC < 100 μg/mL), moderate (100 < MIC ≤ 625 μg/mL) or weak (MIC > 625 μg/mL)^99,100^. In addition, Tamokou et al*.*^101^ proposed new threshold values of MIC for extracts as follow; highly active (MIC < 100 μg/mL), significantly active (100 ≤ MIC ≤ 512 μg/mL), moderately active (512 < MIC ≤ 2048 μg/mL), low activity (MIC > 2048 μg/mL), and not active (MIC > 10 mg/mL). According to these classifications, the activities *M. oleifera* SRRP extract were moderate to significant against *A. tumefaciens* and *P. atrosepticum* and weak to moderate against *E. amylovora*.

Total polyphenols (13.7 g/100 g extract dry weight) and total flavonoids (69.0 g/100 g extract dry weight) were reported from the pods^2,102^. Several phytochemical compounds were identified in different parts of *M. oleifera* including quercetin, ellagic acid, gallic acid and kaempferol^103^.

Revealed to the concentration used, *Salvadora persica* root-bark acetone extract showed inhibition zones (IZs) against *A. tumefaciens* (13.6–18.6 mm), *P. atrosepticum* (15.3–23 mm)^51^. Chloroform leaf extracts from *Lantana camara Duranta plumieri variegata* and *Citharexylum spinosum* showed IZs with the range of 8.3–24.3 mm, 8–13.6 mm, 8–11.6 mm, against *A. tumefaciens*, and 6.6–9.6 mm, 0–9.3 mm, and 9.6–13.6 mm against *P. atrosepticum*, respectively^50^. *Callistemon viminalis* flowers acetone extract observed IZ value 15.0 mm against the growth of *A. tumefaciens*^49^.

*Moringa oleifera* SRRP extract-treated wood showed potential antifungal activity against *F. culmorum* (IFMG 36.88% at concentration 30,000 µg/mL) and *R. solani* (IFMG 51.66% at concentration 30,000 µg/mL). Also, the present results showed that the FMIP against *F. culmorum* was lower than the standard biofungicide Fluconazole (25 μg), which observed IFMG 53.70% and higher than Fluconazole (42.96% against *R. solani*) when applied to wood samples^34^. Previously, different parts of *M. oleifera* plant extracts have been observed to inhibit some phytopathogenic fungi including *Alternata burnsi*, *Aspergillus niger*, *A. paracitic*, *A. flavus*, *Candida Albicans*, *F. oxysporum* and *Trichoderma harzanium*^104^. Comparing to other natural extracts applied to wood samples as biofungicide preservatives, i.e.,* Haplophyllum tuberculatum* whole plant extract with its main compounds resveratrol, kaempferol, myricetin, rutin, quercetin, and rosmarinic acid showed potential antifungal activity against *F. culmorum* and *R. solani* when applied to *Melia azedarach* wood^34,105^. The extracts from *Coccoloba uvifera* with its main compounds of gallic, benzoic, ellagic, and *o*-coumaric acids applied to *Pinus roxburghii* wood observed good activity against *R. solani*, *Botrytis cinerea*, and *F. culmorum*^37^. Flower extract from *Acacia saligna*-treated *M. azedarach* wood, with the presence of quercetin, naringenin, benzoic acid, *o*-coumaric acid, caffeine and kaempferol compounds observed antifungal activity against *F. culmorum*, *R. solani*, and *Penicillium chrysogenum*^26^. An antimicrobial potential activities against *R. solani*, *F. culmorum* and *A. tumefaciens*, were observed as wood-treated with *Musa paradisiaca* peel extract, where the HPLC analysis of the extract identified gallic acid, ellagic acid, naringenin, rutin, and myricetin as main compounds^27^. Furthermore, salicylic acid, rutin, vanillic acid and myricetin were found in *Withania somnifera* fruit extract that showed good wood-biofungicide activity against *F. culmorum* and *R. solani* wood-bactericide against *A. tumefaciens*, *E. amylovora*, and *Pseudomonas cichorii*^106^.

Myricetin which found in the amount of 56.27 mg/100 g extract of* M. oleifera* SRRP, has been previously possessed potential antibacterial activities^107^, also myricetin and rutin were observed potent antifungal agents against *Candida albicans* and *C. parapsilosis*^108^. *A. flavus* and *A. parasiticus* were completely inhibited in terms of their growth and the production aflatoxin by vanillic and caffeic acids at 0.2 mg/mL^109^. Also, phenolic compounds of *Stenoloma chusanum* extract including vanillic acid showed potential antifungal activity^110^.

## Conclusion

As from the present study and commercially, moringa, the fast growing with multipurpose uses, and after obtaining the ripened seed, the seeds-removed pods have been shown some important properties. It acts as a raw material for the production of pulp and paper due to limited wood resources, where the mechanical and physical properties of the produced papersheet were comparable with those reported from the literature from woody and non-woody materials. Also, from the HPLC analysis of phytoconstituents profile, some important phenolic compounds vanillic, benzoic, syringic, and ferulic acids and flavonoid compounds myricetin, naringenin and kaempferol were identified. This study showed the maximizing the utilization of moringa residues in the pulp industry and the production of bioactive chemicals.
