# Author Correction: *Moringa oleifera* seeds-removed ripened pods as alternative for papersheet production: antimicrobial activity and their phytoconstituents profile using HPLC

**DOI:** 10.1038/s41598-021-01035-6

**Published:** 2021-10-26

**Authors:** Mohamed Z. M. Salem, Hayssam M. Ali, Mohammad Akrami

**Affiliations:** 1 Forestry and Wood Technology Department, Faculty of Agriculture (EL-Shatby), Alexandria University, Alexandria, 21545 Egypt; 2 Botany and Microbiology Department, College of Science, King Saud University, P.O. Box 2455, Riyadh, 11451 Saudi Arabia; 3 Department of Engineering, University of Exeter, Exeter, EX4 4QF UK

Correction to: *Scientific Reports* https://doi.org/10.1038/s41598-021-98415-9, published online 24 September 2021

In the original version of this Article Hayssam M. Ali was incorrectly affiliated with ‘Timber Trees Research Department, Sabahia Horticulture Research Station, Horticulture Research Institute, Agriculture Research Center, Alexandria, Egypt’. This affiliation has consequently been removed. The correct affiliation is listed below.

Botany and Microbiology Department, College of Science, King Saud University, P.O. Box 2455, Riyadh, 11451, Saudi Arabia

The original Article has been corrected.

